# Methods, Challenges and Potentials of Single Cell RNA-seq

**DOI:** 10.3390/biology1030658

**Published:** 2012-11-16

**Authors:** Daniel Hebenstreit

**Affiliations:** The University of Warwick, School of Life Sciences, Coventry CV4 7AL, UK; Email: d.hebenstreit@warwick.ac.uk; Tel.: +44-(0)-2476-574457; Fax: +44-(0)-2476-523568

**Keywords:** RNA-seq, scRNA-seq, single cell

## Abstract

RNA-sequencing (RNA-seq) has become the tool of choice for transcriptomics. Several recent studies demonstrate its successful adaption to single cell analysis. This allows new biological insights into cell differentiation, cell-to-cell variation and gene regulation, and how these aspects depend on each other. Here, I review the current single cell RNA-seq (scRNA-seq) efforts and discuss experimental protocols, challenges and potentials.

## 1. Introduction—the Importance of Transcriptional Profiling at Single Cell Resolution

Transcriptome profiling has been a popular tool in molecular biological research for more than a decade. Mostly implemented by microarray technology, it has led to numerous insights and discoveries. These range from cell type specific single factors that were identified in differential expression screens to findings based on large portions of the entire transcriptome, such as disease signatures (e.g., [1]) and the interrelations of epigenetic modifications and gene expression (e.g., [2]).

Following its recent introduction, RNA-sequencing (RNA-seq) [3,4] is rapidly replacing microarrays as the method of choice for the aforementioned endeavors. Besides superior accuracy in the quantification of expression, RNA-seq offers other advantages, such as the possibility to detect novel transcripts, splice variants or allele-specific expression [5]. mRNA-profiling was performed on single cells early [6] and RNA-seq is following suit [7].

The analysis of the transcriptome in individual cells offers a number of advantages compared to cell-averaging experiments. Tissues or other cellular assemblages are heterogeneous even if a single, “traditional” cell type is concerned. This is particularly apparent from the study of immune cell types that are often defined based on the expression of surface markers. Improving experimental technologies reveal continuous expression ranges and fairly unrestricted combinatorial expression of surface markers [8]. This means that boundaries between cell types are blurred and that every individual cell is different. A similar picture emerges with tumors. While tumors have been known to be heterogeneous mixtures of cell types for a long time [9], pioneering studies demonstrate the potential of genome sequencing in individual cells [10]. RNA-seq will thus provide a powerful means to facilitate functional characterization of the differences among the cells in a tumor.

While tumor cell heterogeneity can be attributed to accumulating mutations, even genetically identical cells, under identical conditions, display high variability in their gene and protein expression levels. This is usually referred to as “noise”, defined as variance or standard deviation over mean [11]. A number of studies have probed into the origins and mechanisms of noise and found it to be mostly due to the stochastic effects associated with the low numbers of involved molecules [12].

While standard microarray or RNA-seq experiments yield mean expression levels, distributions in single cells demonstrate that only a negligible portion of cells express mRNAs close to the actual mean levels. Depending on the skewness of the distributions, this could mean that cells that express certain mRNAs at “outlier” levels are functionally important, yet remain undetected by traditional experiments. Knowledge about the shape of the distributions can also be used to understand mechanisms that are involved in transcriptional regulation [13]. 

## 2. RNA-seq—Basic Experimental Procedures

RNA-seq ideally allows the accurate quantification of mRNA expression levels, covers the entire transcript lengths at equal representation at each position, and retains strand information. Depending on the experimental protocol, particularly for single cell RNA-seq (scRNA-seq), these goals are not simultaneously achievable with current methods.

Sample processing for RNA-seq is largely based on traditional molecular biological protocols that were developed decades ago for common assays such as RT-PCR, northern blots or microarrays. The vast majority of RNA-seq methods thus include the basic steps of poly-(A)+ RNA isolation, fragmentation, reverse transcription, and amplification before the actual sequencing takes place.

The selection of poly-(A)+ RNA is usually performed in order to suppress the “loss” of sequencing reads to structural RNAs such as rRNA and tRNA, which represent the bulk of cellular RNA. One disadvantage of this is that certain protein coding RNAs without the poly-(A) tail, such as replication dependent histones, are lost [14]. Total RNA based protocols do exist [15] and the technological advances in sequencing depth could soon render the removal of RNAs other than mRNA obsolete.

The fragmentation step is carried out in order to produce many short RNA or DNA fragments that represent the original transcript. Various methods, including enzymatical, mechanical, and chemical ones have been used successfully. Depending on the exact protocol, the fragmentation serves to optimize the length for the sequencing machine, generates more reads, thus allowing more accurate quantification, and/or is used to reduce positional biases in the transcript representation.

This bias is largely due to reverse transcription that is used to generate the DNA template for PCR or *in vitro* transcription (IVT), which are both popular means to amplify the material. Reverse transcription is necessary, since the suitability of RNA-dependent RNA polymerases [16] for RNA amplification is unclear. Depending on the type of primer that is used, the reverse transcriptase starts polymerizing internally or at the end of the RNA template. In both cases, a bias is induced that is affected by the processivity of the enzyme. The bias induced by internal random priming worsens with a lower enzyme drop-off (although the total yield gets higher), while the opposite is seen when a fixed primer position (e.g., at the transcript 3' end) is used. In the latter case, a roughly exponentially decreasing coverage is expected [17], which is observed experimentally [18].

These biases can be strongly reduced if the fragmentation step is performed on the original RNA [19]. This may not be desirable, however, as it seems likely to damage the RNA and lead to the loss of some of it. Amplification-free RNA-seq might abrogate the need for reverse transcription and thus remove the aforementioned biases (in addition to PCR biases). One effort to that end has been reported as FRT-seq [20], which has not been tested for scRNA-seq.

## 3. ScRNA-seq Strategies

**Figure 1:**
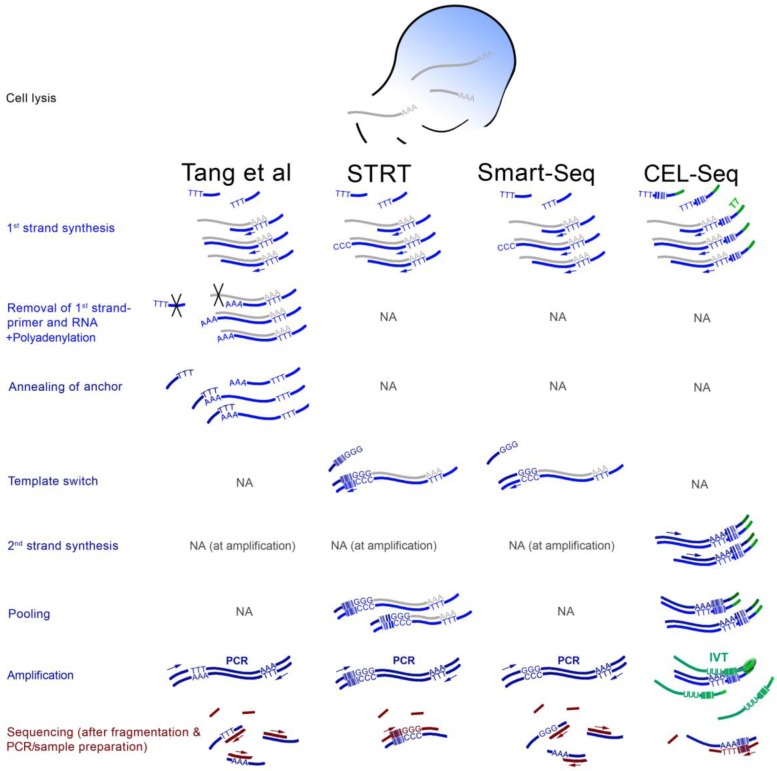
Overview of four scRNA-seq methods. The scheme is a simplification and the focus is the differences between the protocols and the steps that are characteristic to each. The Tang protocol [21,25] includes attachment of adenine nucleotides to the 3' ends of first strands which serve as binding sites for an anchor oligonucleotide, and the RNA and 1st strand primers are removed enzymatically (black crosses). STRT and CEL-seq [23,24,26] include sample pooling based on first strands with barcodes at 5' and 3' regions, respectively, with regards to the original transcripts. CEL-seq uses IVT as first amplification step, which requires direct RNA adapter ligation followed by a second reverse transcription during sequencing sample preparation. Smart-Seq [27] is a simplification of STRT without barcoding. All PCRs are primed from sequences introduced by the flanking oligonucleotides. Arrows indicate primer-initiated DNA polymerization. Nucleotide stretches (such as AAA or TTT) are for illustration only and do not reflect the actual number of nucleotides. The color-coding was chosen to roughly discriminate between the crucial 1^st^ strand-related steps (light blue), protocol-specific steps (dark blue), and the more interchangeable sequencing sample preparation (burgundy).

Four different scRNA-seq strategies have been explored so far (Figure 1). The first study appeared in 2009 [21]. The authors performed reverse transcription directly in the cell lysate primed from a poly-(dT) oligonucleotide containing an anchor sequence on its 3' end. Avoiding a random primer makes it unnecessary to purify poly-(A)+ RNA. Excess primers are digested with exonuclease after which terminal transferase is used to attach poly-A tails to the 3' ends of the first strands. Another poly-(dT) primer with a different anchor is annealed to the poly-(dA) stretch and is used to initiate second strand synthesis. The library is then PCR-amplified from the two anchor sequences, fragmented, and subjected to the sample preparation specific to the sequencing machine. Importantly, here the second strand synthesis begins from wherever the first strand synthesis stops, introducing a strong bias depending on the drop-off of the reverse transcriptase. Fragmentation after PCR further means that the strand information is lost.

Two other scRNA-seq approaches are similar in their usage of the “template-switching” method. This method, also marketed as “Switching mechanism at the 5' end of the RNA transcript” (SMART) [22] exploits the intrinsic property of the Moloney murine leukemia virus (MMLV) reverse transcriptase to add a few non-templated nucleotides (mainly cytosines) to the 3' end of the cDNA first strand [23]. An oligonucleotide with (rG)_3_ on its 3' end (and a suitable 5' sequence that will later serve as primer binding site for PCR amplification) anneals to these nucleotides as soon as the first strand is complete, and provides a template-extension. The RT thus switches from an RNA- to a DNA-template (Figure 1). Importantly, addition of the non-templated nucleotides is most efficient at the 5' end of the RNA transcript [22]. Thus, incomplete first-strands are selected against, in contrast to the terminal transferase-based scRNA-seq.

The first SMART-based scRNA-seq protocol was named “Single-cell tagged reverse transcription” (STRT) [24]. The template-switching oligonucleotides feature a barcoding sequence. Samples can thus be pooled after first strand synthesis and can be sequenced in a multiplex reaction. Since the barcode is located at the 5' ends of the transcripts, only this part will be sequenced after fragmentation (and a purification step). This means that there will be a maximum of one sequencing read for each original transcript, which ideally maps to the corresponding transcriptional start sites.

Although it was published later, the second SMART-based scRNA-seq, “Smart-Seq”, is essentially a simplified version of the first one [27]. No barcoding was used, and fragments were not purified according to their position or orientation. Similar to the Tang protocol [21], oligo-(dT) priming was used directly in the cell lysate to circumvent purification of poly-(A)+ RNA. Although strand-specificity was thus lost, the authors report a markedly improved coverage across the transcript lengths. This protocol, in slightly altered form, was successfully implemented to profile the transcriptome of single neurons [28].

CEL-seq provides the latest addition among the tested scRNA-seq protocols [26]. This strategy is unique in relying on IVT instead of PCR for sample enrichment. The IVT is initiated from a T7 promoter that is introduced with a barcoded oligo-(dT) 1^st^ strand primer. Its advantage lies in the linear mode of amplification, which, unlike PCR, does not exponentially deplete of sequences that are difficult to process [29]. The ability of T7 polymerase to repeatedly bind to the promoter and polymerize means that substantial amplification is achieved in the same reaction, without the need for denaturing and primer re-annealing. CEL-seq is demonstrated to outperform STRT in sensitivity and accuracy [26]. Curiously, the protocol converts the mRNA to DNA, to RNA, and again to DNA (Figure 1).

Common to all four scRNA-seq methods is the usage of two rounds of amplification (including the sequencing technology-specific sample preparation). It should be further noted that the SMART protocol does not lead to better transcript coverage in the sense that the full-length cDNA is synthesized more often. Instead, incomplete cDNAs are less likely to be carried on in the protocol. The downside of this is that transcripts, which could at least have partially contributed to the information gained by the experiment, are discarded.

## 4. Findings from the First scRNA-seq Studies

It is difficult to directly compare the technical performance of the existing scRNA-seq schemes based on their original descriptions, aside from the obvious differences such as strand specificity or transcript coverage. The means to assess performance were largely different in the three works (comparison with microarrays, spike-in controls, titration of the RNA starting mass, correlation with standard RNA-seq, *etc*.) and differences in parameters such as PCR cycle numbers might have strong effects on the final results. A specific effort to evaluate all four methods against each other under identical conditions might be desirable, as it has been done for conventional RNA-seq protocols [30]. To a certain degree, the different methods are modular and allow recombining parts of the protocols. For instance, template switching can be used for 1^st^ strand synthesis in CEL-seq, or barcoding may be used with the Tang protocol.

The potency of scRNA-seq to reveal novel biological insights is explored in the original and follow-up studies of these. One widely used advantage of RNA-seq over microarrays is the possibility to detect splice variants, which has been demonstrated on single cell basis for all three PCR-based scRNA-seq protocols. The Smart-Seq and STRT works further illustrate how global gene expression profiles can be used to categorize cells into traditional cell types, while individual cells might exhibit closer relatedness to cell types other than the one they are classified as [24,27]. The usefulness of CEL-seq is demonstrated for cell lineage analysis in *C. Elegans* [26]. Since the exact number of cells is known with certainty in scRNA-seq experiments, spike-in controls can be used to estimate the absolute levels of mRNA expression. Islam *et al.* use this to reveal a 20-fold higher RNA-content in mouse embryonic fibroblasts (MEFs) compared to embryonic stem cells (ESCs) [24]. In a first application of their scRNA-seq protocol, Tang et al. investigated the transition of inner cell mass (ICM) cells to ESCs in mouse embryos [31]. This revealed a high cell-to-cell variation for genes expressed at intermediate levels, suggesting that they have a higher propensity for dynamic transcriptional regulation among cells of the same traditional cell type. In addition, many probable target genes of pluripotency factors such as Nanog, Sox2, Oct4, or Esrrb appeared to be regulated in a cell autonomous manner [31]. This demonstrates the usefulness of scRNA-seq and the novel biological insights it generates.

## 5. Outlook—Challenges, Future Issues and Emerging Technologies

The first few studies on scRNA-seq have proven the feasibility of this type of experimental assay. The true strength of the method will probably become fully apparent when it is implemented to study the characteristics of distributions of mRNA expression levels among individual cells. This is likely to yield decisive new insights into transcriptional regulation [13].

To fully exploit this potential, several requirements must be met. Precise parameter estimation for mathematical models of transcriptional regulation is usually based on single-molecule fluorescent *in situ* hybridization (smFISH) of at least a few hundred cells each under different conditions (e.g., [32]). This necessitates an upscaling of scRNA-seq, which will be probably most easily implemented by multiplexing as in STRT and CEL-seq. A further issue that is currently unsolved is the quantitative accuracy of scRNA-seq. Spike-in controls will help to understand how sequencing read numbers translate into the original numbers of transcripts. Yet, this does not appear to be a trivial issue, as synthetic RNA seems to behave differently from endogenous RNA in the STRT protocol, for instance [24]. Approaches such as smFISH or NanoString technology provide means to calibrate scRNA-seq based on absolute numbers of endogenous RNA [33,34]. To achieve accurate calibration, this will have to be done for a large number of different types/lengths of mRNA, though.

One means to monitor amplification bias is to introduce a different type of barcode that identifies individual molecules [35]. Multiple occurrences of the same barcode indicate that the corresponding sequenced fragments were derived from only one original molecule. This has highlighted the biases introduced by PCR and has been shown to increase RNA-seq accuracy [36,37]. It has been used only downstream of cDNA production though, and does not protect against complete loss of molecules. It will thus be further beneficial to have mathematical models that describe the sample preparation process, as it is clear that the SMART protocols deplete of incomplete first strands. In addition, CEL-seq probably induces non-trivial biases during the multiple strand conversions. These effects have to be taken account of and thus precisely estimated, if the number of sequencing reads is to be used to determine the exact original transcript numbers.

Another promising strategy is clearly to minimize the number of processing steps. An effort in this vein is direct sequencing technology, as implemented by the Helicos system. This allows sequencing of cDNAs without sample preparation [38] and makes it possible to abolish reverse transcription altogether by direct sequencing of RNA [39,40]. One limitation is the lower number of reads that are currently obtained with this technology [5]. Other upcoming technologies such as the method developed by Pacific Biosciences enable single molecule sequencing and are potentially adaptable for single cell experiments [41,42].

Particularly promising are nanopore sequencing technologies, which represent an entirely new approach. Here, the DNA or RNA strand is threaded through (biological or solid-state) pores of nanometer dimension that are inserted into membranes. Bases that pass through the pores are detected electronically or optically [43].

Further help for sample preparation will be microfluidics systems such as provided by RainDance Technologies or Fluidigm. These have been mainly designed for targeted amplification and highly parallelized PCRs (e.g., [44]), but have considerable potential for adaption for scRNA-seq. Fluidigm notably offers a machine (“C1”) that captures single cells and performs reverse transcription and amplification.
